# Symptomatic Hyponatremia following Bowel Preparation for Colonoscopy: A Case Report

**DOI:** 10.31729/jnma.5039

**Published:** 2020-11-30

**Authors:** Pramesh Sunder Shrestha, Utsav Acharya, Bipin Karki, Rahul Pathak, Subhash Prasad Acharya

**Affiliations:** 1 Department of Anaesthesiology, Maharajgunj Medical Campus, Maharjgunj, Kathmandu, Nepal; 2 Department of Critical Care Medicine, Om Hospital and Research Centre, Chabahil, Kathmandu, Nepal; 3 Department of Gastroenterology, Maharajgunj Medical Campus, Maharjgunj, Kathmandu, Nepal

**Keywords:** *colonoscopy*, *colorectal disease*, *hyponatremia*, *polyethylene glycol*

## Abstract

Colonoscopy is considered a gold standard tool for the diagnostic evaluation of colorectal diseases. Bowel preparation, a pre-requisite for a colonoscopy, usually involves the ingestion of purgatives for the cleansing of the bowel so that visualization is not obscured during the procedure. Commonly used preparations are sodium phosphate-based solutions, sodium picosulphate, and polyethylene glycol. The use of such preparations is associated with electrolyte disturbances, commonly hyponatremia. Hyponatremia is usually seen with sodium phosphate-based solutions and is rare with polyethylene glycol. Symptomatic hyponatremia, however, is rare following bowel preparation and is attributable to other factors as well, such as the age of the patient, non-osmotic release of anti-diuretic hormone, and the procedure itself. In this report, we discuss a case of severe symptomatic hyponatremia observed in a 71-year-old gentleman who underwent polyethylene glycol-based bowel preparation for colonoscopy.

## INTRODUCTION

Colonoscopy is still a gold standard tool for the diagnosis of diseases of the colorectal mucosa.^1^ Cleansing of the bowel is a prerequisite for colonoscopy and is usually done by oral administration of purgatives, commonly sodium-phosphate (NaP) based solutions or polyethylene glycol (PEG) based solutions. Electrolyte abnormalities following bowel preparation is a possibility, especially with NaP based solutions.^2^ As PEG-based solutions are usually reserved for those at risk of developing electrolyte imbalance, PEG is considered relatively safe.^3^ Although electrolyte abnormalities have been reported with PEG-based solutions as well, symptomatic hyponatremia is rare.

## CASE REPORT

A 71-year-old gentleman was transferred to our intensive care unit (ICU) from surgical ward after he was found to be drowsy for the last few hours. He was admitted to the surgical ward the day before, with complaints of loss of appetite for the last six months and passage of blood in stool for one month. There was also a history of significant weight loss for the last six months. He was a known diabetic under the medication and was being managed in the ward with suspicion of malignancy of the large bowel.

**Figure 1 A:**
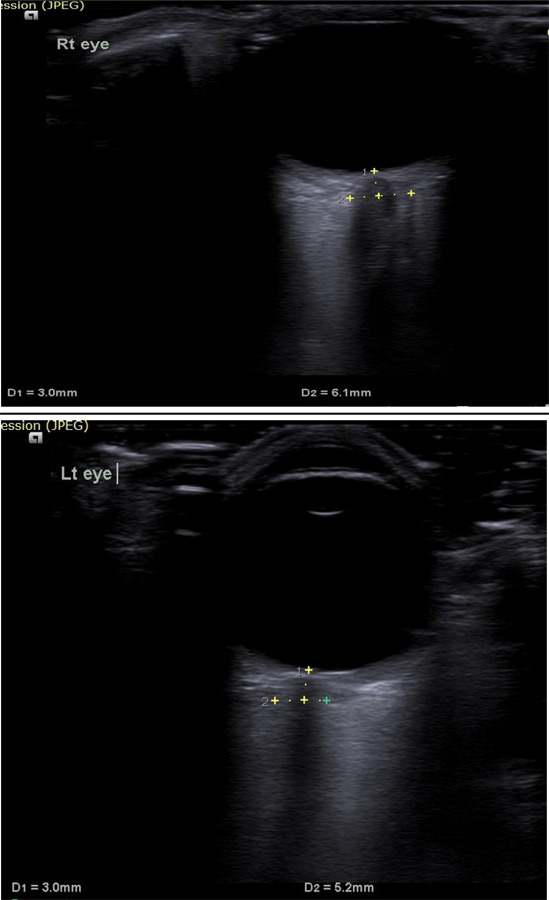
ONSD Right Eye. B. ONSD Left Eye.

On arrival in the ICU, his Glasgow Coma Scale (GCS) score was 10/15 (E_3_V_2_M_5_). Pupils were bilateral equal in size and reactive to light. He was afebrile and there were no signs of meningeal irritation. Reflexes and jerks were normal. There was no history of recent administration of any sedatives. A colonoscopy was performed about four hours back, during which sedated with midazolam. However, the patient had regained full consciousness before he was transferred to the ward. An examination of other systems revealed no abnormalities. Arterial blood gas (ABG) analysis and serum electrolytes were sent among which Magnesium, Phosphate, and Calcium were normal. The ABG was normal. The report of serum electrolytes revealed hyponatremia, with the sodium (Na+) level of 111mmol/L. The blood glucose level was normal. On further evaluation of the history, it was revealed that the patient had ingested around 7-8 liters of overzealous free water overnight with the PEG solution for bowel preparation for colonoscopy. He had presumed extra water would cleanse the bowel and make the procedure easy and more diagnostic. In the ICU patient developed generalized tonic-clonic seizure which was treated with intravenous midazolam and leverecietam was added as anticonvulsant. Optic Nerve Sheath Diameter was recorded which was 6.1mm in Right Eye and 5.1mm in Left Eye (Figures 1a and b).

**Figure 2:**
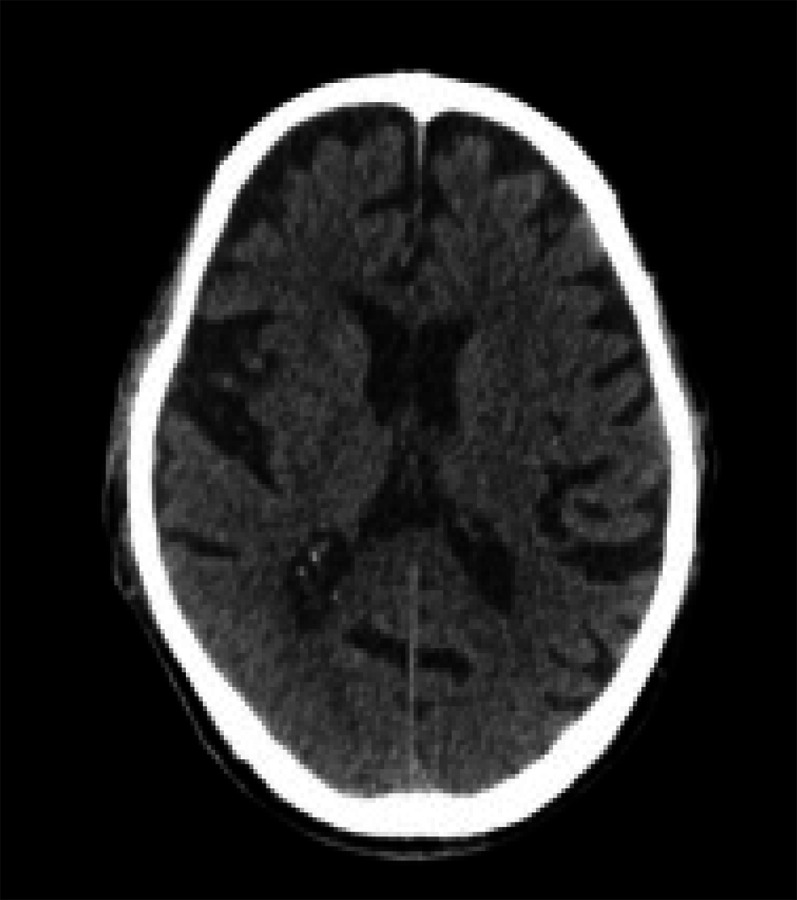
CT scan of the head showing a normal scan with cerebral atrophy.

An urgent computed tomography (CT) scan of the head was done which revealed a normal scan with cerebral atrophy (Figure 2).

**Figure 3:**
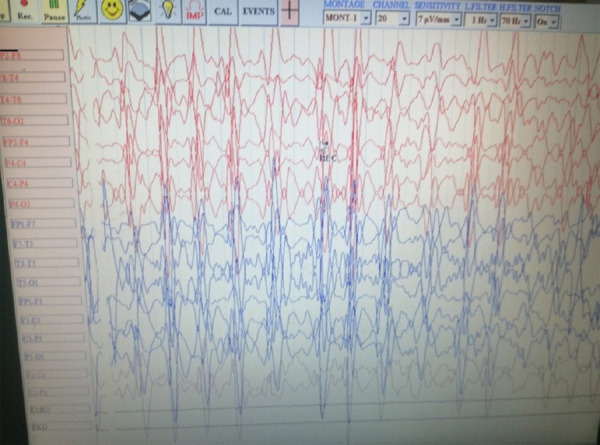
EEG showing seizure activity.

The patient was started on injection of 3% NaCl for treatment of hyponatremia. Electroencephalogram (EEG) was done which recorded seizure activity (Figure 3).

So, levericetam 1g intravenous was reloaded and the dose increased. Sodium gradually increased to 117mmol/L on next but there was no neurological improvement. After 2 days, the sodium increased to 132mmol/L, there was a gradual improvement in neurological status with GCS of E4V5M6. The patient was discharged home after 3 days of hospital stay with normal electrolytes report. Follow up histopathology report was normal.

## DISCUSSION

Hyponatremia is seen in 15-30% of in-patients in a hospital, especially in intensive care units. The etiology is multifactorial but the basic abnormality lies in the body not being able to excrete free water or an excessive intake of free water.^4^ The features range from asymptomatic to severe neurologic manifestations such as seizures, permanent brain damage, coma, and death.^1^

Hyponatremia has been reported in 7.5% of patients undergoing colonoscopy.^5^ The factor most commonly attributable to this is the osmotic shift resulting from the use of osmotically active solutions for bowel preparation. However, studies have shown that although contributory, bowel preparation alone is not the lone factor responsible for electrolyte abnormality. More recently, the role of a non-osmotic release of antidiuretic hormone (ADH), low dietary solute intake, and excessive and rapid free fluid intake have also been considered as contributing factors for hyponatremia associated with colonoscopy.^1^ Non-osmotic release of ADH is usually associated with vomiting, pain, anxiety, stress, and hypoglycemia.^6^ Our patient had an excessive intake of free water the day before a colonoscopy. This, coupled with the age-related reduction in the ability of the kidneys to excrete free water load could have contributed to hyponatremia. As our patient developed features after the procedure, the anxiety and discomfort during the procedure could have triggered a non-osmotic release of ADH.

Whatever the reason, it is very important to keep in mind the possibility of electrolyte imbalance during the preparation of elderly patients for colonoscopy. Preparedness and anticipation will help physicians identify the problem sooner and act promptly so that the neurologic sequelae of hyponatremia can be checked before it progresses to irreversible neurological damage.

## References

[1] Windpessl M, Schwarz C, Wallner M (2017). "Bowel prep hyponatremia" - a state of acute water intoxication facilitated by low dietary solute intake: case report and literature review. BMC Nephrol.

[2] Frizelle FA, Colls BM (2005). Hyponatremia and Seizures After Bowel Preparation: Report of Three Cases. Dis Colon Rectum.

[3] Weir MA, Fleet JL, Vinden C, Shariff SZ, Liu K (2014). Hyponatremia and Sodium Picosulfate Bowel Preparations in Older Adults. Am J Gastroenterol.

[4] Sahay M, Sahay R (2014). Hyponatremia: A practical approach. Indian J Endocrinol Metab.

[5] Gabriel JCM, Muñoz SR, Bertolo JDC (2003). Electrolytic disturbances and colonoscopy : bowel lavage solutions, age and procedure. Spanish J Gastroenterol.

[6] Saradna A, Shankar S, Soni P (2018). Hyponatremic Seizures after Polyethylene Glycol Bowel Preparation: The Elderly at Risk. Am J Ther.

